# The Neolithic site “La Marmotta”: DNA metabarcoding to identify the microbial deterioration of waterlogged archeological wood

**DOI:** 10.3389/fmicb.2023.1129983

**Published:** 2023-03-23

**Authors:** Marzia Beccaccioli, Claudia Moricca, Luigi Faino, Rita Reale, Mario Mineo, Massimo Reverberi

**Affiliations:** ^1^Department of Environmental Biology, “Sapienza” University of Rome, Rome, Italy; ^2^Chemistry Applied to Restoration, A. Galli Academy, Via Petrarca, Como, Italy; ^3^Museo delle Civiltà/Museo Nazionale Preistorico Etnografico “L. Pigorini”, Rome, Italy

**Keywords:** wood biodegradation, amplicon sequencing, erosion bacteria, archeological waterlogged wood, Neolithic

## Abstract

**Introduction:**

The evaluation of biological degradation of waterlogged archeological wood is crucial to choose the conservative and protective treatments to be applied to the wooden material. The waterlogged environmental conditions are characterized by oxygen scarcity, only allowing the growth of adapted microbes capable to degrade the organic wooden material, mainly erosion bacteria and soft-rot fungi. In this work, we characterized and evaluated the biodegradation state and the microbial communities of wooden fragments preserved in storage tanks. These were preserved by waterlogging within the Neolithic village “La Marmotta,” currently found under the Bracciano Lake (Lazio, Italy).

**Methods:**

The waterlogged wood samples were first identified taxonomically with an optical microscope, also allowing an evaluation of their preservation state. The microbial community was then evaluated through the sequencing of Internal Transcribed Spacer sequences for fungi and 16S for bacteria with the Oxford Nanopore Technologies (ONT) MinION platform.

**Results:**

The identified microbial community appears to be consistent with the waterlogged samples, as many bacteria attributable to the erosion of wood and ligninolytic fungi have been sequenced.

**Discussion:**

The reported results highlight the first use of targeted metabarcoding by ONT applied to study the biodeterioration of waterlogged archeological wood.

## 1. Introduction

Wood is a material that has played a key role in the development of mankind. It has and continues to be used as fuel, as a construction material and to make tools and furniture (Gellerstedt, 2009). It is produced by seed-bearing species and its anatomy can be used for the process of identification and classification of tree taxa, both of fresh and archeological specimens (Cartwright, 2015). From a chemical point of view, three major components make up wood cell walls: cellulose, lignin, and hemicellulose. The skeletal matrix is composed of cellulose, while the amorphous hemicelluloses are associated with the cellulose structure. Finally, the amorphous and isotropic lignin encrusts both the hemicelluloses and cellulose (Daniel, 2009).

The preservation of wood in archeological contexts can occur under specific environmental conditions. The most common modality of fossilization in the Mediterranean is charring, meaning the reduction to carbon because of overheating (Renfrew, 1973). Wood can also be preserved by desiccation, in arid environments, or by waterlogging. The latter occurs by the anaerobic conditions caused by continuous wetting. Waterlogged wood absorbs water (up to 700% of its oven-dry weight), becoming swollen and heavy (Pearsall, 2015). Archeological wood from waterlogged contexts can be preserved for an extended time, unlike in oxygenated environments with availability of nutrients, where it is degraded relatively quickly (Jordan, 2001). Preservation of wood by waterlogging occurs in different contexts, such as harbors (e.g., Stagno et al., 2021), peat bogs, pits or lakeshore dwellings (Miksicek, 1987).

Wooden materials are naturally subjected to biodeterioration, defined as any unwanted change in the properties of a material due to the action of living organisms (Hueck, 2001). The most studied causes of wood biodeterioration are xylophagous insects (Coleoptera and Hymenoptera) or fungi. In the first case, the macroscopic damage is mechanical and is easily recognizable by the cavities formed, characterized by regular or irregular configurations (Hueck, 2001). In contrast, fungal colonization degrades the wooden components enzymatically. Ligninolytic, cellulolytic and hemicellulolytic enzymes cause a pathological condition in the plant host with consequent loss of structural coherence in the wood. Symptoms of this pathology are macroscopically visible and classified into three main categories: brown caries, white caries, and soft caries (Blanchette, 2000). Less known is the role of bacteria in the decomposition of wooden materials. Unlike fungi, these can adapt to a wide range of environmental conditions based on the availability of oxygen and nutrients (Blanchette, 2000). Lignin, cellulose, and hemicellulose are susceptible to bacterial attack as they represent an easy carbon resource for all those strains capable of decomposing these substances to obtain carbon (Schmidt and Liese, 1994).

Understanding the fungal and bacteria community living on cultural heritage objects is crucial to preserve them from degrading and it is useful for choosing the best restoration method. During the last years, the application of metagenomic and metataxonomic approaches exploiting the MinION sequencing has attracted the attention of the community of microbiologists involved in the conservation of cultural heritage. The interest on this methodology is related to the ease of use, portable system, reduced costs, and the fast library generation before sequencing. This culture-independent method allows to understand the microbial profile of the artifacts, overcoming the limits imposed by *in vitro* selection.

The MinION sequencing system has been used for the identification of the microbial communities of several cultural heritage materials. This approach can be performed on the whole genome (metagenomics) or using specific regions (metabarcoding); both approaches have been applied to study different cultural heritage materials. The metagenomic approach provides the whole genome amplification and the following sequencing. It has been used on 18th-19th century oil paintings on canvas (Piñar et al., 2020a) and on Leonardo da Vinci’s drawings (Piñar et al., 2020b). Metagenomic analysis produces data useful for studying biodiversity and taxonomic lineages. However, this approach requires heavy computational analysis.

The amplification of targeted sequences (metabarcoding) consists in the amplification of specific regions of fungi, bacteria, or other organisms. This approach was used to study the microbial community of mural paintings in the hypogeum of the *Basilica di San Nicola in Carcere* (Rome, Italy; Grottoli et al., 2020), textiles belonging to funeral accessories from the 17th century (Kisová et al., 2020), and an 18th century wax seal (Šoltys et al., 2020). The application of metabarcoding allowed to use a smaller amount of DNA, simplifying data analysis.

**Figure 1:**
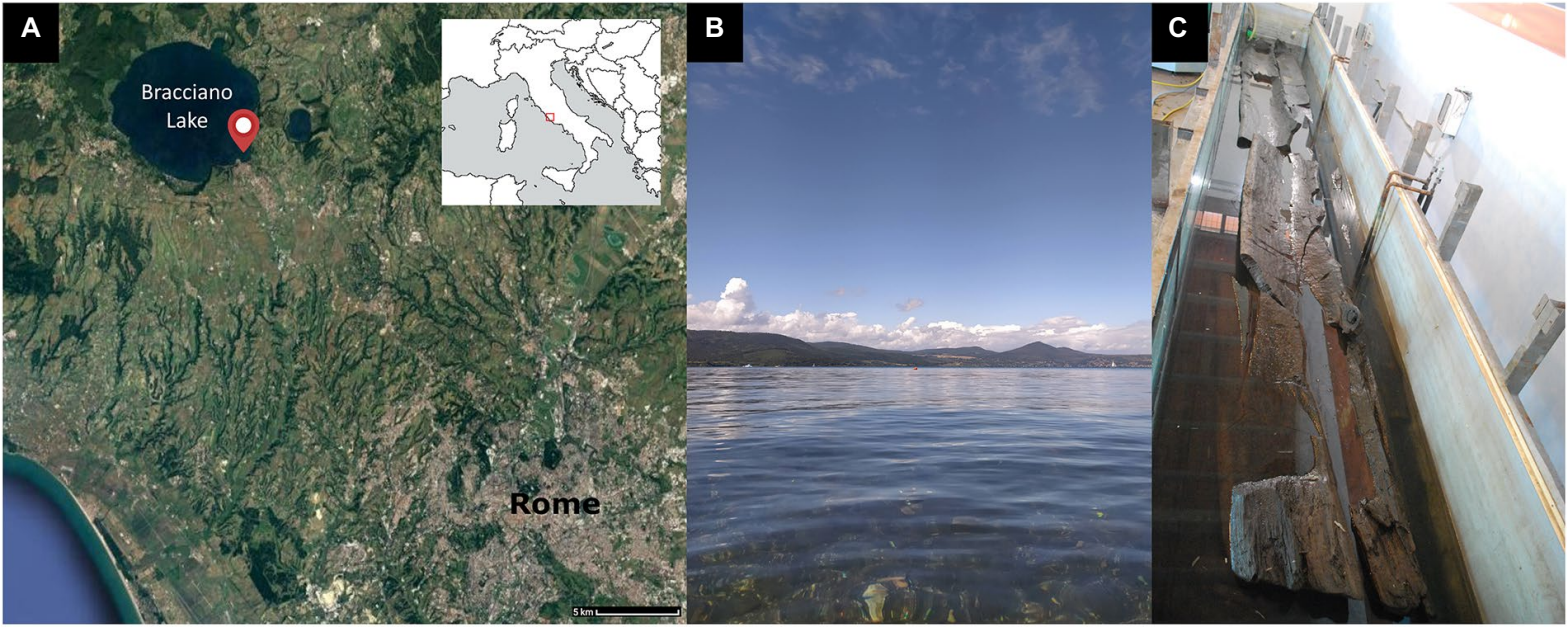
**(A)** The geographical location of the “La Marmotta” Neolithic site with respect to Rome, **(B)** the Bracciano lake, and **(C)** pirogue “Marmotta 5” in the storage tank at the “Anguillara Sabazia Neolithic Visitor Center.”

Metabarcoding or amplicon sequencing is a promising new approach for fungal and bacteria detection and identification. In this paper we report the first application of this method to study the microbial degradation of waterlogged archeological wood. To understand the diversity of taxonomic groups of the Fungi kingdom, DNA barcoding targeted the internal transcribed spacer (ITS) region of the nuclear ribosome (Toju et al., 2012). The bacterial community analysis was based on the amplification of 16S rDNA-gene (Johnson et al., 2019). This study provides a protocol to describe the microbial community of biodeterioration agents related to wood decay in the era of massive DNA sequencing that exploits Oxford Nanopore Technologies (ONT) MinION platform (Grottoli et al., 2020; Santos et al., 2020). Analysis of ONT 16S rRNA and ITS amplicon reads were performed by a custom pipeline. The descripted approach was applied on wooden specimens from the Neolithic village “La Marmotta,” found in Anguillara Sabazia (Rome, Italy; Figure 1A) and currently submerged by the waters of the Bracciano lake (Figure 1B). The site dates to *ca.* 5,700 BC and is one of the most important Early Neolithic sites of the Mediterranean, being acknowledged for the exceptional conservation of organic materials (Mazzucco et al., 2022).

The most important findings at “La Marmotta” are represented by five monossil boats, defined as pirogues. Four of these have been previously subjected to detailed studies and restoration procedures, with the three best preserved ones being on display in the “Luigi Pigorini” National Museum of Prehistory and Ethnography of Rome (Fugazzola and Mineo, 1995; Fugazzola Delpino and Mauro, 2014). The fifth pirogue, also known as “Marmotta 5,” is the last boat to have been recovered (Figure 1C). It is of particular interest as it was abandoned in antiquity while still under construction. “Marmotta 5” was stored at the “Anguillara Sabazia Neolithic Visitor Center” for over 15 years, being subject to scientific studies and conservative interventions since 2020.

## 2. Materials and methods

### 2.1. Sample description and collection

Three waterlogged wood samples from the lacustrine site of “La Marmotta” were for the present study. These include a fragment of the “Marmotta 5” pirogue, brought to light during the excavation campaign of 2006 and since then stored in a big tank in the “Anguillara Sabazia Neolithic Visitor Center.” At the time of our study, the boat was already under consolidation using PEG (polietilenglicole). The fragment that we sampled had detached from the pirogue. The other samples object of our study on microbial community are represented by a beam (F1-MARM) and a pole (F2-MARM), both excavated during the archeological campaign of 1996. Since then, they were stored in a storage tank in the “L. Pigorini” Museum in Rome, where they were kept in a tank filled with tap water at ambient temperature. In May 2021, we manually sampled (using gloves) fragments of approximately 5 cm of length, following the natural fractures of wood. After sampling, all materials were stored in separate containers filled with distilled water and stored in a refrigerator in the Department of Environmental Biology of “Sapienza” University of Rome.

The first step of our analysis concerned a visual evaluation of their conservation state. This was followed by the identification of taxa based on wood anatomy.

### 2.2. Identification of wood taxa

A first analysis of the wood samples was performed using a Leica M205C stereomicroscope (magnification between 7.8x and 160x), ensuring a less invasive approach. Whole wood fragments were placed on the stage plate and oriented according to the anatomical sections of wood (transversal, tangential and radial), allowing an observation of their diagnostic characters. The transverse section (or cross-section) is perpendicular to the direction of plant growth, while the tangential and radial sections are longitudinal. The former of these corresponds to the section tangential to wood rings, while the latter follows the direction of rays. Identification was performed using reference atlases (Schweingruber, 1990; for oaks: Cambini, 1967).

Further observations on the waterlogged samples were done by manually preparing thin section with a razorblade, according to the anatomical sections of wood. The slivers were then places on microscope slides and covered with cover slides. They were observed using a Leica DM750 transmitted light microscope (50-400x magnification), allowing a better assessment of the conservation state of the samples on a microscopic level.

### 2.3. DNA extraction

Samples F1-MARM and F2–MARM were chosen for a metagenomic analysis. The pirogue “Marmotta 5” was excluded from the metagenomic investigation as, at the time of our study, it was already under consolidation. Wood samples were ground with mortar and pestle, and DNA was extracted using the 3C-TAB method (Beccaccioli et al., 2021), based on the use of a three C-TAB buffer [C-Tab I (C-Tab 4%); C-Tab II (C-Tab 10%, NaCl 0.7 M); C-Tab III (C-Tab 1%, Tris–HCl 50 mM)] and Solution 2A (NaCL 2.8 M, Tris–HCL 200 mM at pH 8, EDTA 40 mM at pH 8).

DNA was extracted from 300 mg of wood placed in sterile 2 ml tubes. Extracted DNA was treated with RNase (20 mg/mL, Merck-Sigma-Aldrich, Italy), quality/quantity was checked by agarose gel (1%) and spectrophotometric technology (NanoDrop, ThermoScientific, United States).

### 2.4. DNA amplification by PCR

PCR amplification was performed on 25 ng of DNA of each sample. The broad-spectrum recognition of the bacterial community was performed using universal primers 27-For (5′-GAGATT TGATCCTGGCTCAG-3′) and 1495 Rev. (5′-CTACGGCTACC TTGTTACGA-3′) specific for bacteria 16S rDNA region (Mora et al., 1998). Primers ITS1 (5′-TCCGTAGGTGAACCTGCGG-3′) and ITS4 (5′-TCCTCCGCTTATTGATATGC-3′) have been used to identify the fungal ITS rDNA region (Schoch et al., 2012). Amplification was performed by using Taq polymerase (Bioline, Meridian Bioscience United States), and the PCR products were verified through gel electrophoresis in agarose gel (1%).

### 2.5. Nanospore sequencing

PCR amplicons of each sample were pooled using a ratio of 1:1 (amplicon 16S: amplicon ITS), and purified using AMPure beads. About 45 ng were used for library preparation using the Rapid Barcoding Kit 96 (SQK-RBK 110.96) following the manufacturer’s instructions (Oxford Nanopore Technologies, United Kingdom). The run was performed by Flongle Flow Cell (R9.4.1) on a Mk1B device (Oxford Nanopore Technologies, United Kingdom). Amplicon sequencing run was performed for one o.n. Basecalling and demultiplex was performed by MinKNOW software (v22.08.9) running Guppy software (v6.2.11 + e17754e).

### 2.6. Pipeline to detect the sequences of fungi and bacteria

A custom pipeline was developed to analyze the data generated by Nanopore Ampliseq. In short, to generate fungal and bacterial abundance, reads were trimmed using porechop (v0.2.4) and subsequently aligned against the nt database at the NCBI using BLASTN (v2.12.0). The best alignment for each read was used for read count and plotted using python3 script and ktImportTaxonomy from KronaTools 2.8.1.^1^

## 3. Results

### 3.1. State of conservation

**Figure 2:**
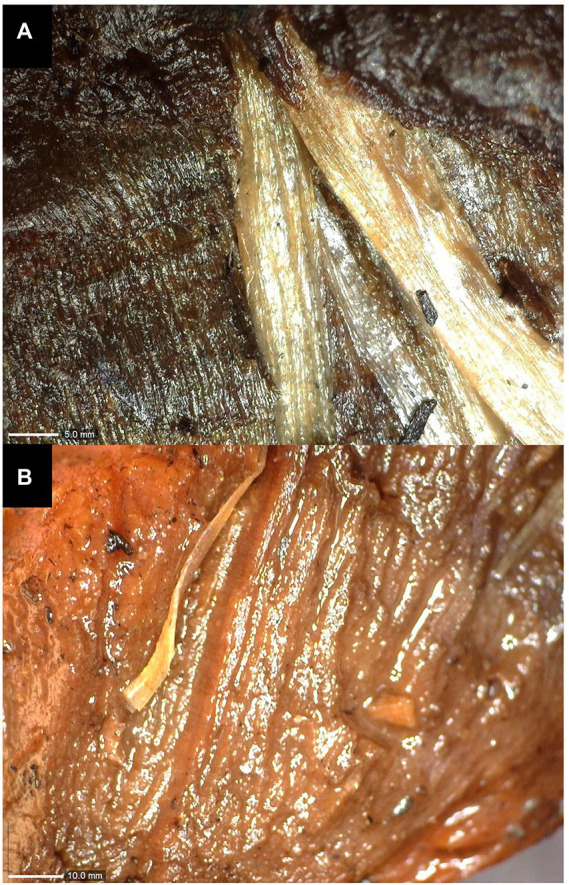
Alterations of waterlogged wood visible to the naked eye: **(A)** leaching of fibers and **(B)** spongy appearance.

**Figure 3:**
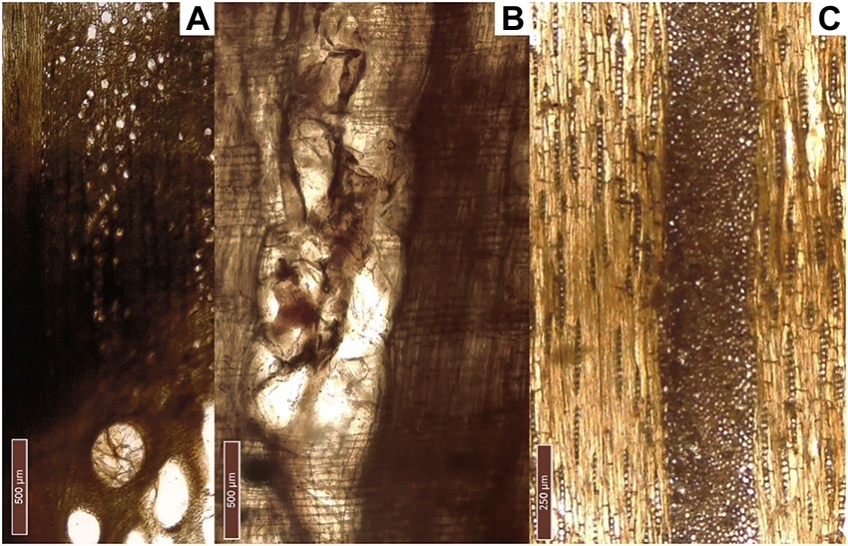
Diagnostic sections of *Quercus* sect. *robur* (F1-MARM): **(A)** transversal section, **(B)** radial section, and **(C)** tangential section.

**Figure 4:**
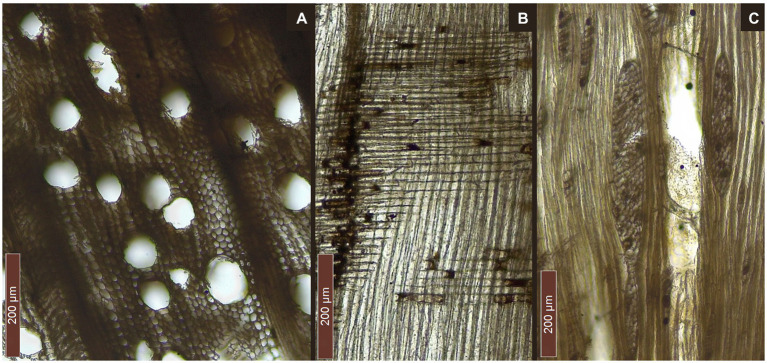
Diagnostic sections of *Fagus* cf. *sylvatica* (Marmotta 5): **(A)** transversal section, **(B)** radial section, and **(C)** tangential section.

The state of conservation of the wooden remains was not only influenced by the environmental conditions of retrieval – lake waters, but also by their storage conditions at the “L. Pigorini” Museum, where they were kept for years in tanks filled with water. Extreme conditions of acidity, the presence of organic and inorganic matter (algae between the fibers and iron salts) have produced varying degrees of alterations that can be observed by naked eye in the form of leaching of the fibers (Figure 2A), spongy appearance (Figure 2B), presence of lesions and extreme fragility. This can be mainly associated to the breaking of cellulose chains, or hydrolysis, of their essential components. While this cannot be observed in the small areas pictured in Figures 3, 4, the extreme fragility of the studied samples and their sponginess were responsible for making it harder to obtain thin sections.

### 3.2. Wood identification

The beam (F1-MARM) was identified as *Quercus* sect. *robur* (Cambini, 1967; Figure 3). This section includes deciduous oaks, such as *Quercus robur* L., *Q. patraea* (Matt.) Liebl., *Q. pubescens* Willd. and *Q. frainetto* Ten., that cannot be distinguished based on wood anatomy. The observed specimen is characterized, in the transverse section, by the presence of a pore ring, composed of big pores with tyloses. Latewood pores are usually solitary, arranged in oblique, dendritic groups. Apotracheal parenchyma is diffuse near the latewood pores, but it also appears in tangential bands. Multiseriate rays are visible by naked eye. Tangentially, it is possible to observe both uniseriate and multiseriate rays. In the radial section homogeneous rays can be seen. Perforation plates are simple. Tyloses can also be observed longitudinally.

The pirogue “Marmotta 5” and the pole (F2-MARM) were identified as being made of *Fagus* cf. *sylvatica* (beech; Figure 4). The transversal section is characterized by diffuse porosity, with numerous pores, often grouped in earlywood and solitary in latewood. Rays are occasionally very large and are distended along growth rings. Tangentially, it is possible to observe both uniseriate and multiseriate rays. Radially, rays appear homogeneous (rarely slightly heterogeneous with square marginal cells). Most perforation plates are simple, but scalariform perforation plates can also be found.

Based on wood anatomy, it is impossible to distinguish between *Fagus* species. However, considering that only *Fagus sylvatica* L. is present in the Italian peninsula, it is probable that the identified samples belong to this species.

### 3.3. Taxonomic profiling of microbial community in the storage tank

In order to identify the microorganisms present in the waterlogged wood bacterial (16S rDNA) and fungal (ITS rDNA) community, amplicons were sequenced by MiniION. After sequencing we obtained 3,706 reads and 5,783 reads for F1-MARM and F2-MARM, respectively. Obtained reads were analyzed by a custom pipeline for taxonomic identification of bacteria and fungi. Obtained reads were then filtered, including only taxa with a frequency of appearance equal to or higher than 1% of the total sum of reads in each sample.

Results showed a restricted range of fungal species when compared to bacterial species: 92.6% in F1-MARM and 95.3% in F2-MARM were bacterial reads, 7.4% in F1-MARM and 4.7% in F2-MARM were fungal reads.

**Figure 5:**
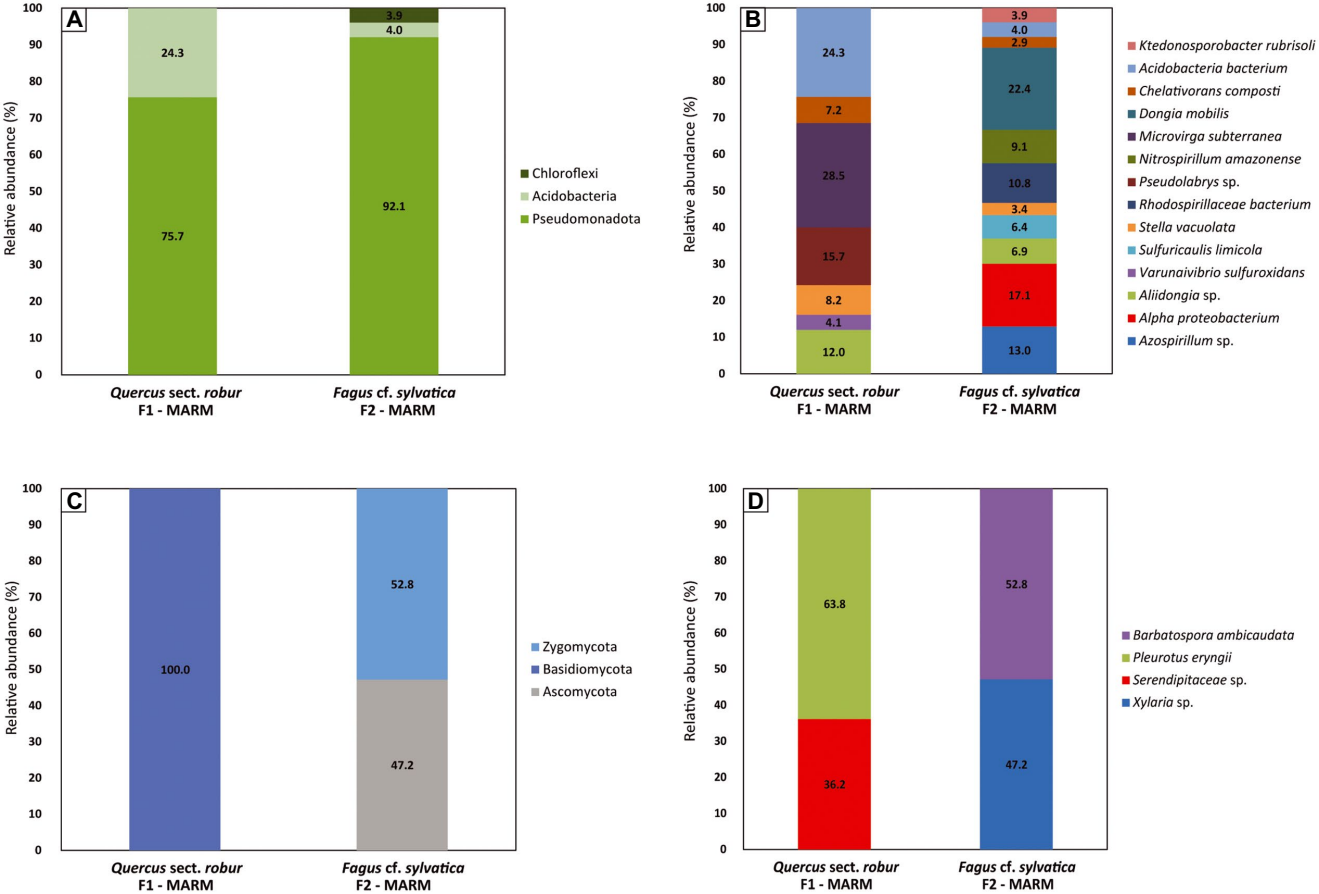
Microorganism diversity of waterlogged archeological wood. **(A)** Bacteria Phylum-level **(B)** Bacteria Genus/Species-level **(C)** Fungi Phylum-level **(D)** Fungi Genus/Species-level relative abundance during the conservation process. Columns represent relative abundance of organisms (Y-axis) per wood sample (X-axis). Values are reported as percentages.

Bacteria reported at phylum-level in F1-MARM were Pseudomonadota (75.7%) and Acidobacteria (24.3%), while in F2-MARM were Pseudomonadota (92.1%), Acidobacteria (4%) and Chloroflexi (3.9%; Figure 5A). In sample F1-MARM (Figure 5B) the bacteria identified belonging to the phylum Pseudomonodota were classified as Alphaproteobacteria, and include: *Aliidongia* sp. (12%), *Stella vacuolata* (8.2%), *Chelativorans composti* (15.7%), *Microvirga subterranea* (28.5%), *Pseudolabrys* sp. (15.7%) and *Varunaivibrio sulfuroxidans* (4.1%). With the phylum Acidobacteria, the predominant species was *Acidobacteria bacterium* (24.3%). In the sample F2-MARM (Figure 5B) the bacteria belonging to the phylum Pseudomonadota detected are classified as Alphaproteobacteria, and include: *Dongia mobilis* (22.4%), *Alpha proteobacterium* (17.1%), *Azospirillum* sp. (13%), *Rhodospirillaceae bacterium* (10.8%), *Nitrospirillum amazonense* (9.1%), *Aliidongia* sp. (6.9%), *Sulfuricaulis limicola* (6.4%), *Stella vacuolata* (3.4%) and *Chelativorans composti* (2.9%). *Acidobacteria bacterium* (4%) was detected for the phylum Acidobacteria and *Ktedonosporobacter rubrisoli* (3.9%) was detected for the phylum Chloroflexota. Comparing the sequencing results of the two specimens, we saw that there were only four strains overlapping between the two sample and that are *Stella vacuolata*, *Chelativorans composti, Aliidongia* sp. and *Acidobacteria bacterium.*

Similarly, fungal community was very much diverse between F1-MARM and F2-MARM. In fact, all fungi reported in sample F1-MARM belonged to the phylum Basidiomycota (100%), while in sample F2-MARM fungal reads belonged to the phyla Ascomycota (47.2%) and Zygomycota (52.8%), showing a wood-selected colonization (Figure 5C). In sample F1-MARM (Figure 5D) the fungi detected included in the phylum Basidiomycota were *Serendipitaceae* sp. (36.2%) and *Pleurotus eryngii* (63.8%). Fungal reads in sample F2-MARM (Figure 5D) corresponded to *Barbatospora ambicaudata* (52.8%) belonging to the phylum Zygomycota, and *Xylaria* sp. (47.2%) belonging to the phylum Ascomycota. All together our results showed that the type of wood (F1-MARM *Quercus* sect. *robur* and *Fagus* cf. *sylvatica*) probably played a crucial role on the microbial colonization.

## 4. Discussion

The identified plant species are coherent with previous studies conducted at the site. Based on the analysis of over two thousand poles at “La Marmotta,” the prevalent taxa were oaks (both deciduous and evergreen), followed by *Laurus nobilis* L. (laurel), *Fraxinus excelsior* L. (ash) and 7 occurrences of *Alnus* sp. (Fugazzola Delpino and Tinazzi, 2010). Furthermore, Rosaceae Maloideae were recorded as the material used to make several sickles in the same site (Caruso Fermé et al., 2021).

Interesting is the use of *Fagus sylvatica* for the pirogue, in contrast with “Marmotta 1” and “Marmotta 2,” respectively made of *Quercus* sp. (oak) and *Alnus* sp. (alder) (*Istituto Centrale per il Restauro*). Nonetheless, this finding is not surprising as beech wood is hard, tough, and easy to work. Beech trees can reach heights of 30–40 m (Packham et al., 2012), making them suitable for the construction of monossil boats.

This is not the first record of this tree being used for such a type of boat, with an example being represented by a Late Bronze Age (1505–1325 BC) pirogue recovered in the Bolsena lake in northern Latium (Calderoni et al., 1996).

Observations performed under the stereomicroscope suggest the preservation of the wood structure overall. It is possible to observe macro-scale degradation phenomena such as thinning and separation of cell wall layers caused by cellulose degradation, which leaves a residual lignin network, where cell walls collapse. Radially, separations and horizontal microfractures can be observed.

**Figure 6:**
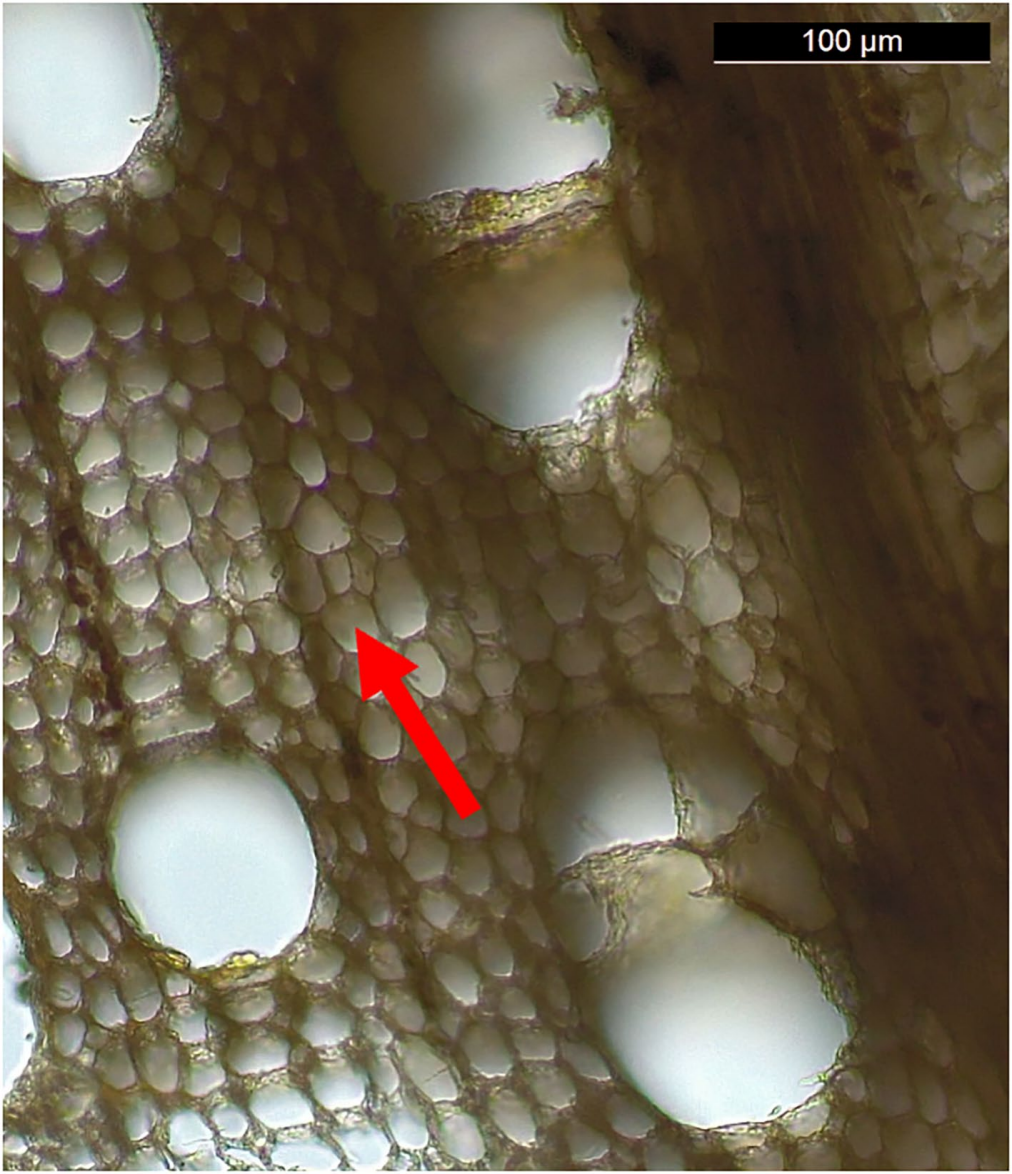
Detail of a thin section of *Fagus* cf. *sylvatica*. The arrow indicates a fiber with a thinned cell wall.

Producing thin sections for detailed anatomical analysis and photographic documentation was complicated, as the razor blades tore the cells apart. This is probably due to anaerobic decay destroying most of the secondary walls in the wood structure (Schweingruber, 1990). Nonetheless, sections of limited areas could be observed, showing an evident decrease in the thickness of fiber cell walls in the transversal section (Figure 6). This could be the result of the action of ascomycetes often associated with soft rot (Osono and Takeda, 2001) and of erosive bacteria (Gao et al., 2018), with a cellulolytic, hemicellulolytic and ligninolytic ability (Yin et al., 2019). To test the degradation capacity of polymers (e.g., cellulose, hemicellulose, and lignin) it is necessary to isolate the biodeteriogens and evaluate the enzymatic activity by *in vitro* assays (Yin et al., 2019). However, this approach underestimates the number of microorganisms due to the limits imposed by *in vitro* selection. Therefore, the development of DNA sequencing technologies has unveiled the variety of uncultured microbes in nature, in particular among aquatic bacteria (Overmann et al., 2017; Pawlowski et al., 2018).

The role of fungal and bacterial colonization on wood decay has already been demonstrated by several studies performed on waterlogged archeological wood from the Nanhai No. 1 shipwreck (Liu et al., 2018). For this typology of study whole genome sequencing (Han et al., 2021) or amplicon sequencing (Liu et al., 2017, 2018; Yin et al., 2019; Jia et al., 2020) have been performed by Illumina platform. In contrast, here we report the first use of MinION amplicon sequencing, which has provided a taxonomic identification capable of assessing fungal and bacterial species related to the soft rot and erosion of waterlogged wood. Even if Nanopore sequencing is used since 2014 for many applications, very few software are available to process Nanopore amplicon sequencing of biological material. Two very well-established software are NanoCLUST and Emu (Rodríguez-Pérez et al., 2021; Curry et al., 2022). Although these two software are largely utilize, they suffer of a limitation: both are developed to handle 16S sequences. However, our analysis was conducted using both 16S and ITS and therefore not applicable with Emu or NanoCLUST. Therefore, we decide to develop a custom pipeline that would be able to use a database made of 16S and ITS sequences to overcome the previously described limitations.

MinION sequencing analysis shows the prevalence of bacteria reads over fungal species, allowing us to hypothesize that they also played a role in the degradation of wood given the anaerobic environment. Our observations suggest that the bacterial community is composed of bacteria belonging to the following phyla: Pseudomonodota, Acidobacteria and Chloroflexi. Pseudomonodota (synonym Proteobacteria) was the dominant bacterial phylum in both samples, 75.7% (F1-MARM) – 92.1% (F2-MARM). Its presence matches the previous studies conducted on waterlogged archeological wood from the Nanhai No. 1 shipwreck (Han et al., 2021). The phylum Acidobacteria was also found (24.3% in F1-MARM and 4% in F2-MARM). The ecological relationship between Acidobacteria and Proteobacteria has already been described. In fact, they are often observed to be intimately associated with each other in the environment (Kielak et al., 2016). The phylum Chloroflexi (3.9%) was encountered only in sample F2-MARM 967049. This phylum was previous encountered in association to cultural heritage, in particular in waterlogged archeological wood (Liu et al., 2018) and on ancient wall paintings (Ma et al., 2015). All the encountered phyla are Gram-negative and rod-shaped, except for *Stella vacuolata* which is star-shaped. The rod-shaped structure is typical of erosion bacteria. Invasion starts from the wood surface, bacteria degrade the cellulose component of the secondary cell wall of which it is rich, transforming it into an amorphous slimy material. In contrast, the middle lamellae, rich in lignin, remain seemingly unaffected, allowing to maintain cell wall integrity in wet conditions. The identification of erosion bacteria is closely related to DNA analysis because they are hard to culture *in vitro* (Björdal, 2012).

In both samples we detected the presence of sulfur-oxiding bacteria, *Varunaivibrio sulfuroxindans* in F1-MARM (Patwardhan and Vetriani, 2016) and *Sulfuricaulis limicola* in F2-MARM (Kojima et al., 2016). The microbial activity in anoxic environment favors the accumulation of sulfur compounds and increases the acidity of the storage environment. These conditions could induce the hydrolysis of cellulose, reducing the mechanical stability of the wood structure (Fors et al., 2014).

The samples show the presence of Rhodospirillaceae (phylum Pseudomonadota), a family of photosynthetic purple bacteria that is able carry out anaerobic respiration (Ferguson et al., 1987). This family includes the genus *Magnetospirillum*, which is believed to play a key role in the degradation of toxic aromatic compounds released from wood during decay, pointing to lignin modification or depolymerization under anaerobic conditions (Van der Lelie et al., 2012). Within family of Rhodospirillaceae, we find *Aliidongia* and *Dongia mobilis* strictly aerobic, and *Stella vacuolate. Dongia mobilis*, present in F2-MARM, was already found associated with a batch reactor for the treatment of malachite (Liu et al., 2010), and its 16 rRNA sequence was recovered in soil (China) and marine costal sediments (India) and Pujalte et al. (2014). *Aliidongia* sp., present in both samples, was already isolated from soil (Chen et al., 2017) and as a colonizer of decaying wood (Mieszkin et al., 2021). *Stella vacuolata*, a star-shaped bacteria present in both samples, was previous recorded in various environments as freshwater, soil, and sewage (Vasilyeva, 1985).

In sample F1-MARM a high percentage of reads match with *Microvirga subterranea* (28%), phylum Pseudomonadota. Reports about this bacteria are really poor, it was only isolated from free-flowing geothermal waters (Kanso and Patel, 2003) and described as thermophilic. Another thermophile was found in both samples, that is *Chelativorans composti* (Kämpfer et al., 2015). Unfortunately, it is really difficult associate the presence of these strains to the wood decay as there is no evidence of them enzymatic activity.

Particular attention goes to the presence of Acidobacteria, a phylum of bacteria most present in fertile soils where plant biomass is an important source of carbon. Acidobacterial genome sequences show the presence of several genes involved in regulating carbon, nitrogen, and sulfur cycles, and those required for degrading different complex polysaccharides (Kalam et al., 2020). This phylum is little known, but genome studies show that some of its strains (subdivision 1) are able to obtain both easily oxidizable carbon from root exudates (such as glucose), and carbon from plant polymers, such as cellulose or xylan, which form the structural components of plants. In fact, they can get carbon through the degradation, by CAZymes, of the d-xylose sugar, component of the hemicellulose contained in the wood, resulting in the decomposition of the latter (Rawat et al., 2012; Dedysh and Damsté, 2018). Although the fungal family of Serendipitaceae is known for its effective promotion of plant growth (mycorrhizae), it is also characterized by the secretion of CAZymes (De Rocchis et al., 2022). The sample F2-MARM show the presence of *Ktedonosporobacter rubrisoli* (phylum Chloroflexi), of which genomic studies revealing a high cellulolytic potential provided by the morphological similarity with the structure of the actinomycetes, widely known in the industrial field for their use in the degradation of plant biomass and its reuse. The presence of enzymes, called CAZymes (Carbohydrated Active Enzymes), capable of performing lysis on polysaccharide components which include endoglucanases, exoglucanses and β-glucosidase, make the class of Ktedonobacteria noteworthy in the conservation field (Zheng et al., 2021), although there is no evidence of direct degradation on submerged wood of archeological nature.

Taxonomic identification of bacteria shows the presence of strains related to the wood decay, and the partial overlap of bacterial species between the two wood specimens suggest specific and targeted colonization at species-level.

Also fungal communities appear more distinct on the base of wood identification: in the sample of F1-MARM encountered only basidiomycetes (*Pleurotus eryngii* and *Serendipitaceae* sp.), while in the sample F2-MARM a zygomycete (*Barbatospora ambicaudata*) and ascomycete (*Xylaria* sp.) are present. Probably, fungal community distribution is affected partly by the decay stage and wood density of its substrate as previous reported in a study conducted on *Quercus* and *Fagus* (Yamashita et al., 2015).

In the sample of F2-MARM we observe an interesting bacterial-fungal interaction with the putative scope of deteriorating the deadwood. The association encountered is among fungi (*Barbatospora ambicaudata* and *Xylaria* sp.) and nitrogen-fixing bacteria [*Azospirillum,* (Steenhoudt and Vanderleyden, 2000) and *Nitrospirillum amazonense* (Schwab et al., 2018)]. Nitrogen availability in deadwood is highly restricted, therefore fungi may take advantage from associations with nitrogen-fixing bacteria for their growth (Gómez-Brandón et al., 2020).

We have examined the archeological wood exposed for a very long time in the storage tanks. Current consolidation practices and musealization have affected the number of samples available for studies, which are statistically low. However, our results provide a method to deeply identify the microbial community and assess the biodegradation status of waterlogged cultural heritage. The condition of the material is always a key issue when considering future storage options.

## 5. Conclusion

Our multidisciplinary study, involving optical microscopy and metabarcoding, has allowed us to properly characterize waterlogged wood fragments from the Neolithic site “La Marmotta” and the degradation that they have undergone.

Despite the good conditions of recovery of the submerged material, a long period before carrying out the consolidation may have led to the appearance of new areas of decay, a risk factor for the integrity of the finds, favoring the proliferation of anaerobic bacteria capable of degrading the main components of wood.

Although the water in the storage tanks was periodically refilled, the results of our analyzes present a number, albeit minimal, of species potentially harmful to organic material, often subjected to biological attack.

We used a qualitative approach to evaluate the biodegradation state and the microbial communities of archeological wood fragments preserved in storage tanks. This study showing a diagnostic process for the determination of the microbial load present in underwater conservation contexts, capable of providing useful information to reduce the danger of biodegradation.

In general, molecular biology associated with sequencing represents a valid cognitive approach, which can be followed by the design of a suitable program for the conservation of artifacts or cultural assets potentially subject to bacterial or fungal attacks.

## Data availability statement

The data presented in the study are deposited in the following repositories: Github (https://github.com/lfaino/Piroga) and NCBI, accession number PRJNA918530.

## Author contributions

MB and CM: conception and design of the study, experimental analysis, data interpretation, and drafting of the manuscript. LF: bioinformatic analysis. LF, RR, MM, and MR: manuscript revision. MR: supervision. All authors contributed to the article and approved the submitted version.

## Conflict of interest

The authors declare that the research was conducted in the absence of any commercial or financial relationships that could be construed as a potential conflict of interest.

## Publisher’s note

All claims expressed in this article are solely those of the authors and do not necessarily represent those of their affiliated organizations, or those of the publisher, the editors and the reviewers. Any product that may be evaluated in this article, or claim that may be made by its manufacturer, is not guaranteed or endorsed by the publisher.

## Footnotes

^1^ https://github.com/lfaino/Piroga
